# Insufficient Milk Supply and Breast Cancer Risk: A Systematic Review

**DOI:** 10.1371/journal.pone.0008237

**Published:** 2009-12-14

**Authors:** Jacqueline M. Cohen, Jennifer A. Hutcheon, Sofi G. Julien, Michel L. Tremblay, Rebecca Fuhrer

**Affiliations:** 1 Department of Epidemiology, Biostatistics and Occupational Health, McGill University, Montreal, Quebec, Canada; 2 Goodman Cancer Centre, McGill University, Montreal, Quebec, Canada; 3 Department of Biochemistry, McGill University, Montreal, Quebec, Canada; Lerner Research Institute, Cleveland Clinic, United States of America

## Abstract

**Background:**

An association between insufficient milk supply, the inability of a mother's breast milk to provide sufficiently for her infant, and breast cancer has been suggested by observations in animal models. To determine if an association has been reported in epidemiological studies of human breast cancer, a systematic review of the literature has been conducted. We also sought to identify the methodological limitations of existing studies to guide the design of any future prospective studies in this field.

**Methodology/Principal Findings:**

PubMed, EMBASE, Web of Science, BIOSIS, and CAB abstracts were searched. We selected any study that (1) assessed breast cancer in association with breastfeeding history and (2) examined the relationship between insufficient milk supply with breast cancer. Seven relevant studies were identified that met both criteria. There was statistically significant heterogeneity among the results which likely reflects clinically significant differences in definitions of insufficient milk supply and reference groups that were used. Among premenopausal women who had experienced insufficient milk supply, odds ratios (ORs) for breast cancer risk ranged from 0.9 to 16.3. Among postmenopausal women, ORs ranged from 0.6 to 6.7. Based on the range of odds ratios obtained in the studies reported in this review, it remains unclear if there is a true association between insufficient milk supply and breast cancer.

**Conclusions/Significance:**

Although some studies have shown a strong positive association, there is no consistent evidence for an effect of insufficient milk supply on breast cancer risk. Exposure definitions are in need of improvement in order to focus on primary insufficient milk supply. Reference groups consisting of women who have successfully breastfed may also introduce positive bias (inflation of the odds ratio) into study results because of the protective effect of prolonged breastfeeding in the control group.

## Introduction

In North America, breast cancer is the second leading cause of cancer-related death in women [1]. Because of the relatively lower percentage of breast cancer cases at younger ages (4% of cases and 2% of all breast cancer deaths among Canadian women ages 20–39 [2]), young women are not typically screened for breast cancer. However, a systematic review of long term survival (10+ years) after breast cancer found that younger age usually entails a more deadly cancer [3]. Further, epidemiological studies have shown that breast cancer diagnosed in close proximity to last birth shows poorer prognosis [4].

Recent evidence from a murine model of breast cancer has suggested there may be a link between breast tumours and insufficient maternal milk supply. Using a well established breast cancer model (in which mammary tumour formation [5] is produced by overexpression of an activated form of the ErbB2 gene), it was observed that mice that are at risk for breast tumours also experience the inability to produce enough milk to support the survival of their offspring.

In many ErbB2 overexpressing cell lines, a protein tyrosine phosphatise, PTP1B, is concomitantly increased in expression [6]. Tremblay's group has recently demonstrated that overexpression of PTP1B in the murine mammary gland causes tumour formation [7]. The phenotype that was observed from mice overexpressing ErbB2 and PTP1B was insufficient mammary glandular development during pregnancy which caused the majority of the offspring to die (10% survival). However, when the transgenic mice overexpressing an activated form of ErbB2 were crossed with a PTP1B knockout, it was observed that mammary gland development was almost fully restored to normal and 85% of the pups survived with weights close to the control litters [8].

These observations in mice have led our group to develop a common cause hypothesis that asserts that misexpression of PTP1B, and potentially of other genes causing defects in breast development, may not only cause difficulties in breastfeeding but could also lead to increased risk for breast tumour formation and cancer progression. If this hypothesis is correct, PTP1B could potentially be a novel biomarker for existing breast cancer or future risk among pregnant or lactating women, especially for those women who experience breastfeeding difficulties caused by the intrinsic incapacity of the breast to produce milk due to inadqequate breast development. However, before a prospective study evaluating this hypothesis in humans is conducted, an understanding of existing work in this field is needed.

Several review articles addressing breastfeeding and breast cancer risk have mentioned an association between insufficient milk supply and subsequent breast cancer [9]–[11]; however it is imperative that a rigorous survey of the literature be carried out on this question. The evidence from such a review can help determine the need for further epidemiologic studies and contribute to incorporating the lessons learned from previous work into future studies.

The objectives of this study were 1) to systematically review the literature that addresses the question: among parous women, is insufficient milk supply during lactation associated with an increased risk of breast cancer? and 2) to identify the methodological or study design limitations of existing studies in order to guide the design of any future prospective studies in this field.

If insufficient milk supply were caused by elevated PTP1B, or other new biomarkers, then this persistent overexpression would most likely cause cancer in the short-term. It seems unlikely from the current evidence [7] that PTP1B could be elevated in the breast tissue for many years without causing a malignancy. Although we cannot exclude the possibility that a link between insufficient milk supply and breast cancer could be associated with postmenopausal breast cancer, we hypothesize that an association would more likely be found among premenopausal breast cancer patients.

## Materials and Methods

### Search Strategy

Our goal was to collect relevant studies that examined the risk of breast cancer in relation to breastfeeding history. We first included studies identified in a systematic review by Berrino et al. done for the WCRF/IACR that encompassed a variety of risk factors for breast cancer, including lactation history [12]. These breast cancer researchers designed a search strategy which was included in a peer-reviewed protocol for their systematic review. The team identified studies from 1966-January 2006 that addressed breast cancer risk in relation to breastfeeding from a variety of electronic databases. We further extended their list of relevant investigations by compiling the most recent studies published during the period of February 2006 to August 8, 2008. Databases included in the search were MEDLINE via PubMed, and EMBASE, ISI Web of Science, BIOSIS, and CAB abstracts via Ovid. The initial search strategy was developed for PubMed and then it was adapted to the other databases. The primary search in PubMed included the following text words in the title or abstract as well as subject headings: (“breast feeding”[MeSH] OR “breast feeding”[tiab] OR “breastfeeding”[tiab]) AND ((mammary AND (cancer* OR neoplasm* OR tumour* OR tumor* OR carcinoma* OR adenocarcinoma*)) OR (Breast AND (cancer* OR neoplasm* OR tumour* OR tumor* OR carcinoma* OR adenocarcinoma*)) OR “Breast Neoplasms”[MeSH]). The searches were limited to English language studies.

### Study Selection

Our search results underwent a primary screen of titles and abstracts to identify any recent study that addressed breast cancer risk in relation to lactation history or more broadly, ‘reproductive factors.’ Any study identified in the primary screen as potentially eligible for inclusion then was assessed in a full-text screen according to predetermined inclusion and exclusion criteria. The inclusion criteria were intentionally broad so that it would be possible to include all evidence that exists, regardless of the study quality, sample size, or other factors. The articles identified in the breast cancer review by Berrino's team were included in the secondary, full-text screen.

The secondary screen of all papers from 1966–2008 that addressed lactation and breast cancer aimed to identify any study that examined an association between insufficient milk supply and breast cancer. For inclusion eligibility, studies must have provided odds ratios (ORs) and 95% confidence intervals (CIs) for estimates of risk of breast cancer associated with insufficient milk supply or data necessary for calculation of unadjusted ORs and 95% CIs. We limited our review to English language studies, and excluded editorials, letters to the editor, conference abstracts, review articles, and meta-analyses were excluded. Studies were also excluded if there was no measure of the relationship between the risk factor and breast cancer reported in the journal article.

### Data Abstraction

Relevant data were independently extracted by two investigators (J.M.C. and J.A.H.) through use of a data extraction form that was tailored for the review and improved via several pilot extractions. Collected data include basic study characteristics such as location and time period, sources of study subjects, age groups included, and other information about study design, including case definitions and participation rates. To elucidate the information that is important for judging study quality, we included a section based on the STROBE statement [13], where each paper could be assessed according to specific items defined in the STROBE statement checklist (http://www.strobe-statement.org/Checklist.html). Finally, results from each study were recorded. These included raw data and crude ORs as well as adjusted ORs, when available, and the potential confounders that were adjusted for in each study.

Extracted data were compared and disagreements were resolved by consensus. Agreement on several predetermined items was assessed to determine inter-rater reliability: Table 1 depicts these specific questions and items. The investigators specified that the raw data to be recorded should reflect the comparisons that were utilized in the authors' analyses, even when data for multiple reference groups were sometimes available.

**Table 1:** Items used to assess inter-rater reliability for data extraction.

	Questions and items
1	Did the authors state any specific objectives?
2	Did the authors give eligibility criteria and sources and methods of case ascertainment and control selection? Did they give the rationale for choice of cases and controls?
3	Did the authors clearly define exposures and outcomes?
4	Potential confounders (list of check boxes)
5	For each variable, did the authors give sources of data and details and methods of assessment?
6	Did the authors describe all statistical methods, including those used to control for confounding?
7	Did the authors report the number of individuals at each stage of the study?
8	Did the authors indicate the number of study participants with missing data?
9	Raw data for 2×2 tables and adjusted odds ratios

### Study Quality Assessment

Study quality was assessed according to the items identified in the STROBE statement for reporting of observational studies. An item that assessed interviewer blinding was added to the adapted checklist as it is an important quality factor in case-control studies. All items were assessed on a yes/no basis, except for case ascertainment. Quality was assessed subjectively by addressing what were considered by the reviewers to be the most important items related to study quality. These items include participation rate, mean time between diagnosis and interview, reporting of sample size calculation, breast cancer assessment method, interviewer blinding, reporting of missing data, and quality of exposure definition which was addressed based on previous research regarding breastfeeding difficulties.

### Statistical Analysis

Crude ORs and 95% CIs were calculated for the association between insufficient milk supply and breast cancer for each study according to the raw data presented in the report. Heterogeneity between studies was assessed with the I^2^ test, a measure of variability related to heterogeneity as opposed to chance. Values for the I^2^ test range from 0–100%; 0–25%, >25–50%, and >50–75% correspond to low, moderate and high heterogeneity [14]. Meta-analysis was performed using a random-effects model to pool study estimates of effect. Heterogeneity was calculated for estimates of pre- and postmenopausal breast cancer risk separately and meta-analysis was used to pool effect estimates for pre- and postmenopausal breast cancer separately. Meta-analysis was only performed if there were three or more studies available for comparison.

After examining the studies to be included, it became apparent that the use of different reference groups slightly altered the research question and therefore, the conclusions that could be drawn from the results. Due to the comparisons with multiple reference groups, studies were stratified based on three broad comparison groups of parous women: 1) successfully breastfed, 2) unsuccessful at breastfeeding for reasons other than insufficient milk supply, and 3) never breastfed. Due to concerns of unadjusted confounding, meta-analysis was not performed on the data comparing women with insufficient milk supply to those women who never breastfed. Meta-analyses of crude and adjusted results were performed separately. Statistical analyses were performed using STATA/SE 10.0 software (STATA, College Station, TX, USA).

## Results

**Figure 1:**
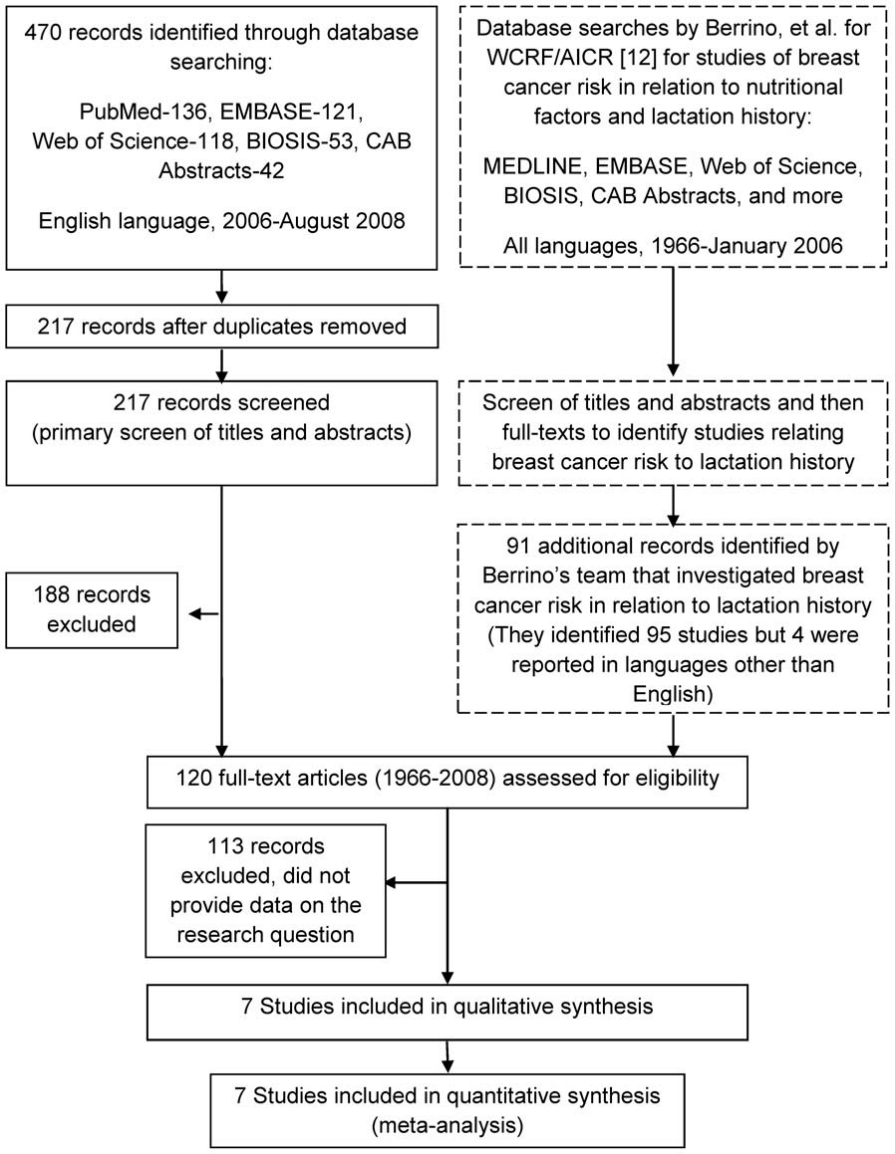
Flow diagram for study selection. Studies from 1966-Jan. 2006 that addressed an association between breastfeeding history and breast cancer were obtained from a systematic review of breast cancer that addressed various risk factors. To obtain studies from Feb. 2006-present, we developed a search strategy that was based on that which was employed by the existing review. All studies from 1966-present were assessed for inclusion in this systematic review. Dashed lines surround the work that was previously published by Berrino, et al.

The combined searches of Berrino et al. and our group (i.e. 1966–2008) yielded 120 articles for the full-text secondary screen (Figure 1). Seven studies were identified in this secondary screen that provided estimates of the effect of insufficient milk on breast cancer risk (Table 2) [15]–[21]. The primary reason for exclusion of studies during the full-text screen was that they did not provide data on our primary research question. Few studies asked subjects why they had discontinued breastfeeding or if they had experienced difficulties breastfeeding. Three studies were excluded in the secondary screen which reported in their methods that they had asked study subjects about reasons for breastfeeding cessation in the interview, but did not report any data related to insufficient milk supply or breastfeeding difficulties in general [22]–[24].

**Table 2:** Characteristics of studies investigating an association between insufficient milk supply and breast cancer.

Author	Year of Publication	Country	Study Period	Outcome (type of breast cancer)	Age (years)	N total
Shema [15]	2007	Israel	2005	premenopausal and postmenopausal	30–75	**640**
Newcomb [16]	1999	U.S.A.	1992–1994	postmenopausal	50–79	**1323**
Freudenheim [17]	1997	U.S.A.	not available	premenopausal and postmenopausal	40–85	**511**
Brinton [18]	1995	U.S.A.	not available	premenopausal	<45	**169**
Newcomb [19]	1994	U.S.A.	not available	premenopausal and postmenopausal	<75	**7454**
Yang [20]	1993	Canada	1988–1989	premenopausal and postmenopausal	<75	**1182**
Byers [21]	1985	U.S.A.	1957–1965	premenopausal and postmenopausal	40–84	**1012**

* note that all are case-control studies.

For data extraction, the average number of items that were in agreement was 7.3 out of the 9 pre-specified items (81%). Reviewers discussed disagreements and they were resolved by consensus. Agreement on raw data necessary for calculation of crude ORs and the reported adjusted ORs was 100%.

### General Characteristics of Studies

The crude and adjusted ORs for the risk of breast cancer among women with insufficient milk supply are summarized in Table 3 (premenopausal breast cancer) and Table 4 (postmenopausal breast cancer) according to the reference group selected in the study. Table 3 and Table 4 also provide more specific details about the reference groups that were used for each comparison as well as the various definitions of insufficient milk supply that were employed in each study.

**Table 3:** Results of studies assessing an association between insufficient milk supply and premenopausal breast cancer; stratified by reference group (a. studies comparing women who experienced insufficient milk to parous women who breastfed successfully, b. breastfed unsuccessfully for other reasons, c. never breastfed).

Study	N cases / N controls	Age	Definition of Insufficient Milk Supply	Crude OR (95% CI)	Adjusted OR (95% CI); Adjusted for…	Notes About Comparison Group
**a.**						
Shema 2007	68/128	30–75	Unsuccessful attempt at breastfeeding or quitting before 1 month because of insufficient milk	16.3 (3.5–150)		Breastfed for 1–12 months and 12+ months combined
Freudenheim 1997	81/110	40–85	Reported as the reason for cessation of breastfeeding within 1.5 months of any birth	1.2 (0.4–3.0)	0.9 (0.3–2.4); Age, parity, education, FHBC, history of BBD*, age at menarche, at first birth	Lactated and did not experience insufficient milk after any birth
Newcomb 1994	556/1150	<75	Reported experience of insufficient milk within the first 3 months of first or second birth	1.0 (0.8–1.3)	1.0 (0.8–1.3); Age, parity, age at menarche, fist birth, first lactation, history of BBD, FHBC, BMI	It is unclear how long the comparison group breastfed for
Yang 1993	178/211	<75	Breastfed unsuccessfully (<1 month) and reported the reason as insufficient milk	3.2 (1.4–8.0)	3.1 (1.4–6.7); Age, parity	Breastfed for ≥2 months
Byers 1985	66/288	40–84	Reported as the reason for cessation of breastfeeding after the first birth	2.2 (1.2–3.9)	2.10; Age	Successfully lactated for any length of time and then stopped for reasons other than insufficient milk supply
**b.**						
Brinton 1995	96/73	<45	Reported as the reason for breastfeeding for <2 weeks	1.5 (0.7–3.6)		Reported breastfeeding <2 weeks for reasons other than insufficient milk
Yang 1993	41/17	<75	Breastfed unsuccessfully (<1 month) and reported the reason as insufficient milk	1.0 (0.3–3.7)		Breastfed unsuccessfully (<1 month) and reported any other reason
**c.**						
Shema 2007	50/34	30–75	Reported unsuccessful breastfeeding attempt or reported quitting breastfeeding before 1 month because of insufficient milk	6.2 (1.3–59.5)		Lifetime duration of breastfeeding = 0 months
Yang 1993	96/76	<75	Breastfed unsuccessfully (<1 month) and reported the reason as insufficient milk	2.2 (0.9–5.8)		Never lactated

* FHBC = family history of breast cancer BMI = body mass index (kg/m2) BBD = benign breast disease.

**Table 4:** Results of studies assessing an association between insufficient milk supply and postmenopausal breast cancer; stratified by reference group (a. studies comparing women who experienced insufficient milk to parous women who breastfed successfully, b. breastfed unsuccessfully for other reasons, c. never breastfed).

Study	N cases / N controls	Age	Definition of Insufficient Milk Supply	Crude OR (95% CI)	Adjusted OR (95% CI): Adjusted for…	Notes About Comparison Group
**a.**						
Shema 2007	123/321	30–75	Unsuccessful attempt at breastfeeding or quitting before 1 month because of insufficient milk	6.7 (3.2–14.6)		Breastfed for 1–12 months and 12+ months combined
Newcomb 1999	685/638	50–79	Experience of insufficient milk within the first 3 months of the first 3 pregnancies	1.1 (0.8–1.3)	1.2 (0.8–1.4); Study site, age, parity, FHBC*, age at menopause, BMI*, education, duration of lactation	It is unclear how long the comparison group breastfed for
Freudenheim 1997	140/180	40–85	Reported as the reason for cessation of breastfeeding within 1.5 months of any birth	0.7 (0.4–1.2)	0.6 (0.3–1.2); Age, parity, education, FHBC, history of BBD*, age at menarche, at first birth	Lactated and did not experience insufficient milk after any birth
Newcomb 1994	2603/3145	<75	Reported experience of insufficient milk within the first 3 months of first or second birth	0.9 (0.8–1.0)	0.9 (0.8–1.0); Age, parity, age at menarche, fist birth, first lactation, menopause, history of BBD, FHBC, BMI	It is unclear how long the comparison group breastfed for
Yang 1993	404/389	<75	Breastfed unsuccessfully (<1 month) and reported the reason as insufficient milk	1.1 (0.7–1.8)	1.1 (0.7–1.8); Age, parity	Breastfed for ≥2 months
Byers 1985	172/486	40–84	Reported as the reason for cessation of breastfeeding after the first birth	1.1 (0.9–2.1)	1.62; Age	Successfully lactated for any length of time and then stopped for reasons other than insufficient milk supply
**b.**						
Yang 1993	70/63	<75	Breastfed unsuccessfully (<1 month) and reported the reason as insufficient milk	1.1 (0.5–2.2)		Breastfed unsuccessfully (<1 month) and reported any other reason
**c.**						
Shema 2007	93/83	30–75	Reported unsuccessful breastfeeding attempt or reported quitting breastfeeding before 1 month because of insufficient milk	1.9 (0.8–4.3)		Lifetime duration of breastfeeding = 0 months
Newcomb 1999	884/861	50–79	Reported experience of insufficient milk within the first 3 months of the first 3 pregnancies	1.1 (0.9–1.4)		Did not nurse for reasons other than insufficient milk
Yang 1993	195/213	<75	Breastfed unsuccessfully (<1 month) and reported the reason as insufficient milk	1.3 (0.8–2.2)		Never lactated

* FHBC = family history of breast cancer BMI = body mass index (kg/m2) BBD = benign breast disease.

Exposure to insufficient milk supply was entirely self-reported. While most studies specified the post-partum time period for which insufficient milk supply could be considered the cause of breastfeeding cessation, Byers' did not. Many women may mistakenly report physiologic declines in milk supply several months after delivery as insufficient milk supply if the time period is unspecified. The definition employed in the Newcomb studies may also include some of these women. It would be preferable to restrict the time period for which insufficient milk supply can be reported, for example for the first 1 month after delivery, as a woman who is unable to provide sufficient nutrition to her infant will most likely not continue breastfeeding for much longer. Brinton's definition of the exposure identified in the first two weeks after delivery is probably too limited. Many of these women could have had other explanations for their breastfeeding difficulties and did not explore the root cause as intensively as others who were more persistent in their attempts to successfully breastfeed.

**Figure 2:**
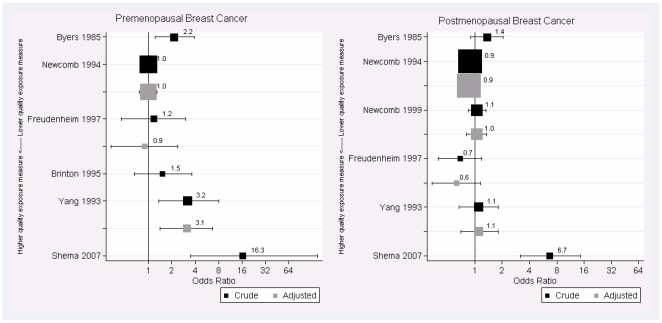
Odds ratios and 95% confidence intervals from studies ordered by quality of exposure definition. Forest plots of the odds ratios (ORs) and confidence intervals (CIs) for the risk of premenopausal breast cancer (left plot) and postmenopausal breast cancer among women who experienced insufficient milk supply compared to women who breastfed successfully, or in the case of Brinton et al, stopped breastfeeding before 2 weeks for reasons *other than* insufficient milk supply. Note that high quality exposure definitions correspond to lower values on the y-axis.

Figure 2 presents forest plots showing the crude and adjusted ORs for the risk of breast cancer among premenopausal women (left) and postmenopausal women (right) who experienced insufficient milk supply as compared to successful breastfeeding, except in the case of Brinton et al. where this reference group was not available. The studies are ranked roughly in order of the quality of the exposure definition, with the highest quality exposure definitions towards the bottom of each plot. We observe that as the definition of insufficient milk supply becomes more focused on the small percentage of women who cannot successfully breastfeed because of inadequate mammary gland development, the ORs for the risk of breast cancer increase.

Brinton et al. identified premenopausal women who had stopped breastfeeding within 2 weeks of delivery [18]. They compared those who stopped because of insufficient milk to women who stopped for other reasons. From the raw data that they presented, we calculated the OR to be 1.5 (95% CI: [0.7, 3.6]). Yang et al.'s data allowed for a similar comparison. We used their raw data to compare women who breastfed unsuccessfully (<1 month) because of insufficient milk to women who were unsuccessful for other reasons [20]. The OR for this comparison was 1.0 (95% CI: [0.3, 3.7]) for premenopausal cases of breast cancer and 1.1 (95% CI: [0.5, 2.2]) for postmenopausal breast cancer.

### Premenopausal Breast Cancer

Studies that compared premenopausal women who had experienced insufficient milk supply to those who had successfully breastfed had crude ORs ranging from 1.0 to 16.3 (Figure 2). Two studies obtained confidence intervals that included the null value of the OR and the other three studies' confidence intervals all exceed the null value, suggesting a possible effect. These results were highly heterogeneous; the I^2^ was 78% (95% CI: [46, 91%]), indicating that most of the variability is due to heterogeneity rather than chance. It is clear just by looking at the forest plot that the results are not consistent, but suggest a possible association. When adjusted estimates of the association between insufficient milk and premenopausal breast cancer were examined, ORs ranged from 0.9 to 3.1, and only Yang's study (which obtained an adjusted OR of 3.1) reported a 95% CI that excluded the null value [20]. These results were also highly heterogeneous; the I^2^ was 73% (95% CI: [11, 92%]). According to the random effects models, the pooled estimate of the crude OR was 2.0 (95% CI: [1.0, 3.9]) and the adjusted OR was 1.4 (95% CI: [0.7, 2.9]).

### Postmenopausal Breast Cancer

Studies that compared postmenopausal women who had experienced insufficient milk supply to those who had successfully breastfed found crude ORs ranging from 0.7 to 6.7, and only the 95% CI from Shema's study which reported an OR of 6.7 excluded the null value [15]. These results were highly heterogeneous; the I^2^ was 84% (95% CI: [67, 92%]). When adjusted ORs were compared for postmenopausal breast cancer, heterogeneity as measured by I^2^ was low, and adjusted ORs ranged from 0.6 to 1.6. The I^2^ was 24% (95% CI: [0, 88%]). All of the CIs for the adjusted ORs included OR = 1, suggesting no effect. According to the random effects models, the pooled estimate of the crude OR was 1.2 (95% CI: [0.9, 1.7]) and the adjusted OR was 0.9 (95% CI: [0.8, 1.1]).

## Discussion

### Main Findings

This systematic review assessed the literature on the risk of breast cancer after experience of insufficient milk supply and has revealed that there has been interest in the question, but studies to date have not brought about any firm conclusions because of heterogeneity in reference groups and exposure definitions. Misclassification seems to be a large problem in these studies due to imprecise or vague definitions of insufficient milk supply. In these studies, the percentage of cases reporting insufficient milk supply ranged from 7–61%. They reported a wide range of results, with estimates of the effect of insufficient milk supply on breast cancer risk ranging from null effects to an odds ratio over 16.

### Interpretation of Between-Study Differences

The use of different reference groups was an important source of heterogeneity among studies in this review. Byers, Shema, and Yang's studies all found that insufficient milk supply was a significant risk factor for premenopausal breast cancer [15], [20], [21]. The comparison groups in each of these studies were women who breastfed successfully for various lengths of time. While not all studies have reported protective effects [25], [26], meta-analysis has demonstrated a reduced risk of breast cancer among women who breastfed their infants [12]; therefore, any *harmful* effect of insufficient milk supply observed could also be explained as a *protective* effect of prolonged breastfeeding in the reference group consisting of women with a sufficient milk supply. Women who never breastfed may provide a more appropriate comparison group; however, women who choose not to breastfeed their children may have an overall less healthy lifestyle and this may introduce bias (confounding) into the results of the association between insufficient milk supply and breast cancer.

Brinton and Yang both chose comparison groups that seemed to be a good compromise between avoiding bias due to the protection afforded by breastfeeding and avoiding unmeasured confounders among women who chose not to breastfeed their infants [18], [20]. Yang's comparison group comprised women who intended to breastfeed and then stopped because of factors other than insufficient milk supply before one month had elapsed since delivery [20]. Brinton made a similar comparison, however chose women who stopped breastfeeding at less than two weeks [18]. Although these comparisons were interesting, these women are rare and therefore limit sample sizes and the power to detect significant associations.

In addition to the heterogeneity of reference groups, the lack of a clear and consistent definition of insufficient milk supply in these studies was also a cause of concern. Insufficient milk supply may be caused by a variety of factors and has repeatedly been cited in the literature as a common reason that women stop breastfeeding [27], [28]. Iatrogenic causes and mismanagement of breastfeeding such as strictly scheduled feeding times and infrequent nursing are among the most common causes of insufficient milk supply [28]. Insufficient mammary glandular tissue, a primary cause of insufficient milk supply, is a rare phenomenon. Based on a prospective study of 319 healthy, motivated, primiparous women with healthy, term infants who received intensive intervention with a lactation consultant, it was estimated that 4 percent of the cases of insufficient milk supply were attributable to a primary problem of the breast due to the lack of sufficient glandular tissue available for milk production [29], [30]. Since this cause of insufficient milk supply most closely resembles the phenotype observed in mice with overexpression of PTP1B [8], it is important that studies examining a link between insufficient milk supply and breast cancer focus on this group of women who suffer from an organic problem of the breast, as opposed to insufficient milk resulting from a potentially modifiable factor.

In the studies reviewed, however, the definitions of insufficient milk supply utilized were often non-specific. According to Byers' definition, a woman could report having experienced insufficient milk supply at any time after her first birth. Women have often misinterpreted the physiologic decreases in milk supply that occur over time to be symptoms of insufficient milk supply. The use of this exposure definition will most likely result in many more exposed subjects than would be predicted based on the reported prevalence of this problem. Indeed, 61% of premenopausal cases and 42% of premenopausal controls in Byer's study reported experience of insufficient milk supply [21]. Although our hypothesis would suggest a higher rate of insufficient milk supply among breast cancer cases than in the general population, we would still expect a much lower proportion than that which was reported from this study. According to Brinton's exposure definition, a woman could report having experienced insufficient milk supply as a reason for quitting breastfeeding in the first two weeks postpartum [18]. Use of this definition will probably lead to the inclusion of women who have experienced delayed lactogenesis but not necessarily persistent insufficient milk supply; when women stop breastfeeding before two weeks it is likely that this is due to very early breastfeeding troubles, therefore, there is also a possibility that the exposure will be over-reported according to this definition. The best definitions were in Shema and Yang's studies (Table 2) which both focused on the first month after birth [15], [20].

The use of non-specific definitions of insufficient milk supply likely lead to important misclassification of the exposure. If both cases and controls equally misclassified themselves as having experienced insufficient milk supply, it is possible that the true association between insufficient milk supply and breast cancer is stronger than that which was found in some of these studies. However, as it is common in case-control studies for cases to over report exposures which they think may be involved in the causal pathway of their disease, it is also possible that differential misclassification could have led to bias towards a positive association.

### Limitations

There were some limitations in this review. Only English studies were included for the database search for 2006–2008. It is unlikely that this introduced a meaningful amount of bias. In the Berrino review, which covered the longest time (from 1966–2006) few studies in other languages were identified and were then excluded in our review process. It was unfeasible to include unpublished data and therefore the possibility for publication bias exists. Because this association was often not the main focus of the included papers, the problem may be important as the results may not have been presented if there was an otherwise uninteresting result. However, based on the studies reviewed here, it does not appear that only positive findings are being published. Only one reviewer was involved in the search and selection of studies, which is another potential limitation of this review, but the majority of studies included in the secondary screen were derived from a systematic review in which the studies had been selected in duplicate.

Meta-analysis is not conventionally carried out in cases where heterogeneity statistics reveal a great deal of variability in study findings, as was observed among the included studies. However, studies addressing the risk of breast cancer in premenopausal women obtained either null or positive findings. We chose to calculate pooled estimates despite heterogeneity, because it was likely that the tests were significant because of widely different magnitudes of the positive effect estimates as opposed to differences in the direction of the effect estimates. The strength of evidence provided by these meta-analyses is admittedly weak because of small sample sizes and widely varied estimates of the association between insufficient milk supply and breast cancer; however, we felt it was important to provide a summary of the effect according to the evidence presented in this review.

### Recommendations for Future Research

In future studies of premenopausal breast cancer, a more specific definition of insufficient milk supply should be used. Interview questions should attempt to identify women with problems breastfeeding whose problems arose at the level of the breast and not from mismanagement of breastfeeding or hormonal imbalance. Follow-up questions should be designed so as to narrow the focus on women who intended to breastfeed but could not do so successfully without supplementing with formula due to poor infant weight gain. It may also be useful to rule out breastfeeding difficulties that were due to the infant's inability to effectively transfer milk from the breast.

A major concern for the design of future studies is that both the exposure and the outcome are rare events among premenopausal women. In a case-control study of women with breast cancer, it is unlikely that many of these women would have experienced primary insufficient milk supply due to its low incidence rate, requiring a large sample size. Nevertheless, case-control studies evaluating breast cancer risk may be the most effective approach as multiple risk factors may be examined in one study and cohort studies are less efficient for rare events.

### Conclusions

No consistent convincing evidence of a link between insufficient milk supply and breast cancer was found. Despite heterogeneous findings of individual studies, a summary of studies comparing women with premenopausal breast cancer with women who successfully breastfed for various lengths of time, however, indicated significantly increased risks of breast cancer among women who experienced insufficient milk supply. More research is needed to determine if a true effect exists, while taking into consideration the importance of the choice of comparison group and the definition of insufficient milk supply. The focus of future research should be on women who do not produce enough breast milk and whose problems cannot be alleviated with current therapies or changes in breastfeeding behavior.
